# Efficacy and safety of EGFR inhibitor gefitinib in recurrent or metastatic cervical cancer: a preliminary report

**DOI:** 10.1007/s12032-023-02070-1

**Published:** 2023-06-13

**Authors:** Abhishek Krishna, M. Sathya, S. Mukesh, M. S. Athiyamaan, Sourjya Banerjee, Johan Sunny, Challapalli Srinivas, Dilson Lobo, Bharat Sai Makkapatti, Vaishak Jawahar

**Affiliations:** 1 Department of Radiation Oncology, Kasturba Medical College, Mangalore, India; 2 Department of Radiation Oncology, Mysore Medical College and Research Institute, Mysore, India

**Keywords:** Gefitinib, Cervical cancer, EGFR, Metastatic

## Abstract

There has been growing interest in the use of epidermal growth factor receptor inhibitors in various cancers. The study was conducted to evaluate the efficacy and safety of gefitinib as a monotherapy in patients with recurrent or metastatic cervical cancer. Patients with cervical carcinoma who experienced locoregional recurrence or distant metastases either at presentation or after definitive combined chemoradiotherapy or postoperative radiotherapy were enrolled. Gefitinib was administered orally at a dose of 250 mg/d to eligible patients. Treatment with Gefitinib was continued until disease progression, intolerable adverse effects were developed, or consent was withdrawn. Clinical and radiological investigations were used to verify the disease response. Toxicity was graded according to the National Cancer Institute Common Terminology Criteria for Adverse Events version 5.0. The study enrolled 32 patients who met the eligibility criteria. Thirty patients were available for the analysis. The majority of the patients included in the analysis had FIGO stage IIIB disease at their initial presentation. The median follow-up time was 6 months (3–15 months). Two patients (7%) had a complete clinical response, 7 patients (23%) had a partial response, 5 patients (17%) showed a stable disease and 16 patients had progressive disease (53%). The disease control rate was 47%. The median PFS was noted to be 4.5 months and the 1-year PFS was 20%. None of the individuals experienced toxicity of grade 3 or higher. All toxicities were managed conservatively. The study suggests that gefitinib may be a promising therapeutic option for patients with advanced cervical cancer who have limited treatment alternatives.

## Background

Cervical cancer is an important health concern across the globe, with an estimated 6 lakh new cases and 3,41,000 deaths in 2020 alone [1]. According to the latest data from Globocan 2020, India accounted for 106,208 new cases and 57,778 deaths in 2020, contributing to 5.5% of all new cancer cases and 6.7% of cancer-related deaths in the country [1, 2]. Cervical cancer is the second most common cancer among women in India and is one the leading cause of death in Indian women aged 15–49 years [1]. The high incidence of cervical cancer in India is attributed to the lack of awareness and access to screening programmes, constrained healthcare infrastructure, and a relatively high prevalence of risk factors such as human papillomavirus (HPV) infection, poor menstrual hygiene, and limited access to advanced healthcare services [3].

The treatment of choice for locally advanced cervical cancer is concurrent chemoradiation with platinum complexes [4]. Despite receiving chemoradiation, 30 to 50% of patients with locally advanced tumours are at risk of recurrence or metastasis [5–7]. The overall prognosis for recurrent and metastatic cervical cancer patients is grim, presenting a considerable clinical challenge to oncologists [6, 7]. Moreover, treatment options for this subset of patients are limited, and typically, palliative in nature. To address this, several chemotherapy regimens have been investigated, including platinum-based regimens [8–10]. However, the response rates to these regimens remain modest at 20–30%, with a median overall survival (OS) of less than10 months [9, 10].

There has been an ever-growing interest in the use of epidermal growth factor receptor (EGFR) inhibitors in various cancers in the recent years [11, 12]. EGFR is a transmembrane protein that is involved in various cellular signalling pathways and plays a crucial role in the regulation of cell proliferation, differentiation, division, and survival [12, 13]. Overexpression or activation of EGFR has been linked to the development of several malignancies, including lung, head and neck, and cervical cancer [14–18].

Gefitinib is a first-generation EGFR tyrosine kinase inhibitor (TKI) that is widely used in the treatment of lung cancer [14, 15]. It acts by inhibiting the activity of the EGFR tyrosine kinase, which prevents downstream signalling pathways from being activated, inhibits cell cycle progression, inhibits epithelial–mesenchymal transition, and induces apoptosis in cervical cancer cells leading to inhibition of tumour cell growth and proliferation [12, 19, 20].

In cervical cancer, the overexpression of EGFR has been associated with a poorer prognosis [21, 22]. Studies have evaluated the use of gefitinib in the treatment of cervical cancer, both as monotherapy and as a combination with other agents [18, 23–25]. In a phase II study, gefitinib demonstrated modest activity as a single agent in patients with recurrent or metastatic cervical cancer, with 20% of patients having a stable disease [18]. Another study investigated the use of gefitinib in combination with cisplatin and paclitaxel in patients with advanced cervical cancer, reporting an overall response rate of 30% and a median progression-free survival of 4 months [24]. Furthermore, most studies have focused on the use of EGFR inhibitors in combination with other treatments, such as chemotherapy or radiation therapy, and only a very few studies have investigated the use of EGFR inhibitors as a monotherapy in cervical cancer. This study was conducted to evaluate the efficacy and safety of gefitinib as a monotherapy in patients with recurrent or metastatic cervical cancer.

## Materials and methods

This was a single-arm, prospective study conducted at a tertiary care hospital in India. Patients with cervical carcinoma who developed locoregional recurrence (LRR) or distant metastases (DM) either at presentation or after definitive combined chemoradiotherapy or postoperative radiotherapy were enrolled. Informed consent was obtained from all participants.

The study involved patients who satisfied the following inclusion criteria: (1) having locally recurrent or disseminated metastatic disease that was not amenable to curative surgical or re-irradiation, (2) deemed unsuitable for active chemotherapy due to low Karnofsky performance score (<60), impaired renal function as a result of underlying pathology, and concomitant comorbidities, (3) exhibiting disease progression despite salvage chemotherapy, and (4) experiencing significant chemotherapy-induced toxicity that rendered continuation of the treatment unfeasible.

Gefitinib was administered orally at a fixed of 250 mg every day to eligible patients. Treatment continued till disease progression, intolerable adverse effects developed, or consent was withdrawn. Patients were monitored monthly, with clinical examination at each visit and a Magnetic Resonance Imaging (MRI) scan or a computed tomographic (CT) scan performed when deemed necessary. Clinical and radiological investigations were used to verify disease response. Response evaluation was conducted using the Response Evaluation Criteria In Solid Tumours version 1.1 [26]. Toxicity was graded according to the National Cancer Institute Common Terminology Criteria for Adverse Events (CTCAE) version 5.0 [27].

The main objective of this investigation was to determine the overall response rate (ORR), which was defined as the percentage of patients who achieved a partial or complete response to the treatment. In addition, secondary endpoints were assessed, including progression-free survival (PFS) and safety and tolerability of the treatment. PFS was defined as the interval between enrolment and the date of either disease progression, death, or the final follow-up, whichever occurred first. To calculate survival, the Kaplan–Meier survival method was used, and SPSS version 22 was utilized for statistical analysis.

## Results

### Patient characteristics

The study enrolled 32 patients who met the eligibility criteria between November 2020 and October 2021. The patients had either recurrent or metastatic cervical carcinoma. Two patients among the 32 were lost to monthly follow-up and hence were excluded from the analysis.

The median age of patients evaluated in the study was of 58.5 years. Majority of the patients (30%) had FIGO stage IIIB disease at their initial presentation. All patients received initial treatment with either radiotherapy and/or chemotherapy. Twenty-three patients received chemoradiation as their initial treatment. 2 patients received only radiation as their primary treatment due to comorbidities. Five patients who were metastatic at presentation received chemotherapy upfront. The patient characteristics are given in Table 1.

**Table 1:** Patient characteristics

Patient details	*n* = 30 (%)
Age	
Median (years)	58.5
Histology	
Squamous cell carcinoma	30 (100%)
Initial FIGO stage	
I	0 (0%)
IIA	0 (0%)
IIB	2 (6.7%)
IIIA	5 (16.7%)
IIIB	9 (30%)
IIIC1	5 (16.7%)
IIIC2	2 (6.7%)
IVA	2 (6.7%)
IVB	5 (16.7%)
Initial treatment	
CCRT	23 (76.6%)
Only RT	2 (6.7%)
Chemotherapy	5 (16.7%)
Site of recurrence/metastasis	
Local	3 (10%)
Regional nodes	12 (40%)
Locoregional	3 (10%)
Distant	12 (40%)
Salvage therapy before Gefitinib	
Chemotherapy	27 (900%)
Palliative Radiotherapy	3 (10%)
Reasons for ineligibility for further therapy prior to initiating Gefitinib	
Toxicity	7 (23.4%)
Disease Progression	14 (46.6%)
Poor Performance status (KPS < 60)	9 (30%)

*FIGO* International Federation of Gynaecology and Obstetrics, *CCRT* concurrent chemoradiotherapy, *RT* radiation therapy, *KPS* Karnofsky performance score

The predominant site of recurrence following prior chemoradiotherapy in patients was observed to be the regional lymph nodes (Table 1). Of the cohort, 27 individuals underwent salvage chemotherapy prior to initiating gefitinib therapy. Disease progression and deteriorating physical performance status were the primary contributors to ineligibility for further chemotherapy and transition to gefitinib therapy.

### Disease response and survival

**Fig. 1:**
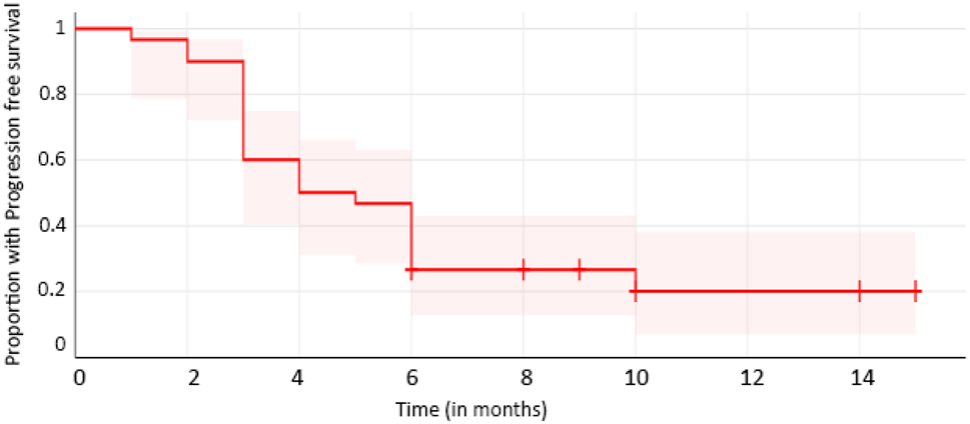
Progression free survival of the patients

Patients were followed up from November 2020 till February 2022 in this preliminary analysis. The median follow-up period was 6 months (3–15 months). Two patients (7%) had a complete clinical response, seven patients (23%) in the research had a partial response, five (17%) patients had stable disease, and sixteen patients had advancing disease (53%). The overall response rate was 30%. The disease control rate, as defined by patients with complete, partial, or stable disease, was 47%. The median PFS was found to be 4.5 months, with a 1-year PFS of 20% (Fig. 1).

### Toxicities

The toxicity was within acceptable levels. None of the patients experienced grade 3 or higher toxicity. There were no deaths because of adverse effects of gefitinib. The most common toxicity was skin rashes seen in 9 patients, but none of them had a grade ≥ 3 or higher skin rash. The second most frequent toxicity noted was diarrhoea, which was seen in four patients (Table 2).

**Table 2:** Frequency and Pattern of toxicity

	Grade 1 or 2 *n* = 30 (%)	Grade 3 and above *n* = 30 (%)
Fatigue	2 (6.7%)	0 (0%)
Nausea	1 (3.3%)	0 (0%)
Vomiting	3 (10%)	0 (0%)
Skin Rash	9 (30%)	0 (0%)
Diarrhoea	4 (13.3%)	0 (0%)
Any	19 (64.4%)	0 (0%)

## Discussion

Cervical cancer is a significant public health concern globally, and systemic chemotherapy with platinum and taxanes is currently the standard of care for majority of the patients with recurrent or metastatic disease [4]. However, many patients experience disease progression or severe toxicity during their chemotherapy, which limits their treatment options. The EGF receptor, which is expressed by various forms of cancer, has been identified as a promising anti-cancer target. In Asian females, non-smoking individuals with adenocarcinoma, EGFR mutations are common, and enhanced response to gefitinib, an EGFR inhibitor, has been reported in this patient population [15]. Although there is no strong evidence, most Indian women are historically non-smokers; hence, individuals with cervical cancer may have EGFR mutations frequently [24].

Gefitinib is an orally accessible drug with ease of administration, making it a promising therapeutic alternative. Unlike intravenous chemotherapy, which requires frequent hospital visits, gefitinib only needs to be taken once a day. Moreover, gefitinib is less expensive than chemotherapy drugs, which can help alleviate the financial burden on patients and their families.

Few studies have been conducted to investigate the efficacy and safety of gefitinib in cervical cancer. Sharma et al. treated patients with advanced cervical cancer who had failed first-line chemotherapy were treated with gefitinib [24]. The median time to progression was 4 months, and the median overall survival was 5 months. The most common adverse events were rash and diarrhoea, which were mild to moderate in severity. The authors concluded that gefitinib had limited activity as a single agent in this patient population but suggested that it might have a role in combination with other agents. A phase II study by Goncalves et al. evaluated the efficacy of gefitinib in advanced cervical cancer and noted that 20% of the patients experienced stable disease with a median duration of 111.5 days [18]. The most common adverse events were nausea, vomiting, and fatigue, which were mostly grade 1 or 2 in severity. Our study investigated the potential efficacy of gefitinib as a treatment option for advanced cervical cancer. We observed a PFS of 4.5 months and a 1-year PFS of 20%, which is consistent with prior studies of gefitinib in this patient population.

Because recurrent and metastatic cervical cancers are usually treated palliatively, the quality of life is a major consideration for these patients. In our study, we found that the symptoms of nine patients improved significantly after gefitinib treatment. However, as no formal quality of life evaluation was conducted, additional research is needed to further investigate the potential effect of gefitinib on quality of life. In addition to these clinical trials, several preclinical studies have investigated the mechanism of action of gefitinib in cervical cancer. EGFR overexpression has been identified in various types of cervical cancer, and preclinical studies have shown that inhibition of EGFR signalling can lead to decreased cell proliferation, migration, and invasion in cervical cancer cell lines [13, 20].

Another advantage of gefitinib is its low incidence of side effects. In our study, all observed side effects were manageable, and no participants dropped out of the trials due to toxicity. These findings are consistent with prior studies of gefitinib, which have reported mild to moderate toxicities [18, 24]. A potential advantage of gefitinib over traditional chemotherapy is its ability to selectively target cancer cells without affecting normal cells. Unlike chemotherapy, which can cause damage to healthy cells leading to adverse side effects, gefitinib specifically targets and inhibits the EGFR pathway that is often overexpressed in cancer cells, making it a more targeted therapy with potentially fewer side effects.

In addition, the use of gefitinib in cervical cancer treatment may also have implications for patients in low-resource settings, such as many parts of the developing world [24]. Cervical cancer is one of the leading causes of deaths among women in these countries, and treatment options are limited by factors such as cost and accessibility [28]. Gefitinib is relatively affordable compared to traditional chemotherapy, and its oral administration makes it more convenient for patients who may not have easy access to healthcare facilities for frequent intravenous treatments. While the efficacy and tolerability of gefitinib are promising, further studies are needed to optimize its use in the treatment of advanced cervical cancer. For example, future studies could investigate the combination of gefitinib with other anti-cancer agents to improve its therapeutic effect. Additionally, the optimal dosage and duration of gefitinib treatment need to be determined.

Despite the promising results, several questions remain regarding the use of gefitinib in cervical cancer. While EGFR overexpression is common in cervical cancer, not all patients may have tumours that are dependent on EGFR signalling. Identification of biomarkers that predict response to gefitinib therapy could help select patients who are most likely to benefit from treatment. Second, the optimal dosing and scheduling of gefitinib in combination with other agents have not been determined. While several clinical trials have evaluated the combination of gefitinib with chemotherapy or radiation therapy, the most effective regimen remains to be determined. Finally, the long-term safety and efficacy of gefitinib in cervical cancer patients have not been fully evaluated. Further follow-up of patients who have received gefitinib therapy is needed to determine the potential impact of the drug on overall survival and quality of life. Further studies should also investigate the potential predictive biomarkers of gefitinib response, such as EGFR mutation status or other biomarkers, which could help identify the patients who are most likely to benefit from gefitinib treatment.

One potential limitation of our study is the small sample size. As a single-centre study, our findings may not be generalizable to other populations or settings. Among the 30 enrolled patients, 14 had documented disease progression before initiating Gefitinib, while the remaining patients did not exhibit progression at the time of starting treatment. While we attempted to minimize the confounding factors, we recognize the inherent challenge in attributing the subsequent response solely to gefitinib in patients who have received salvage chemotherapy prior to initiation of gefitinib. Additionally, our study primarily focused on response rates and progression-free survival (PFS) limiting the assessment of overall survival and comprehensive clinical outcomes. Larger multicentre studies with diverse patient populations are needed to confirm our results and determine the optimal patient population and treatment regimen for gefitinib in advanced cervical cancer.

## Conclusion

The study suggests that gefitinib may be a promising therapeutic option for patients with advanced cervical cancer who have limited treatment alternatives. Its oral accessibility, ease of administration, low cost, and manageable side effects make it an attractive alternative to traditional chemotherapy. Additional research is needed to evaluate the potential effect of gefitinib on overall survival and quality of life in this patient population.

## Data Availability

All data, models, or code generated or used during the study are available from the corresponding author by request.

## References

[1] Sung H, Ferlay J, Siegel RL, Laversanne M, Soerjomataram I, Jemal A (2021). Global Cancer Statistics 2020: GLOBOCAN estimates of incidence and mortality worldwide for 36 cancers in 185 countries. CA Cancer J Clin.

[2] Hull R, Mbele M, Makhafola T, Hicks C, Wang SM, Reis RM (2020). Cervical cancer in low and middle.income countries (Review). Oncol Lett.

[3] Juneja A, Pandey A. A survey on risk factors associated with cervical cancer undernutrition among children under 2 years of age View project Estimation of Malaria Burden in India View project. 2023. https://www.researchgate.net/publication/8925144.

[4] Vale C, Tierney JF, Stewart LA, Brady M, Dinshaw K, Jakobsen A (2008). Reducing uncertainties about the effects of chemoradiotherapy for cervical cancer: a systematic review and meta-analysis of individual patient data from 18 randomized trials. J Clin Oncol.

[5] Lancet SW-T, 2003 undefined. Cervical cancer. 2023. https://www.sciencedirect.com/science/article/pii/S0140673603137786.

[6] Beadle BM, Jhingran A, Yom SS, Ramirez PT, Eifel PJ (2010). Patterns of regional recurrence after definitive radiotherapy for cervical cancer. Int J Radiat Oncol Biol Phys.

[7] Kim TH, Kim MH, Kim BJ, Park SI, Ryu SY, Cho CK (2017). Prognostic importance of the site of recurrence in patients with metastatic recurrent cervical cancer. Int J Radiat Oncol Biol Phys.

[8] Kumar L, Harish P, Malik PS, Khurana S (2018). Chemotherapy and targeted therapy in the management of cervical cancer. Curr Probl Cancer Mosby.

[9] Movva S, Rodriguez L, Arias-Pulido H, Verschraegen C (2009). Novel chemotherapy approaches for cervical cancer. Cancer.

[10] Monk BJ, Sill MW, McMeekin DS, Cohn DE, Ramondetta LM, Boardman CH (2009). Phase III trial of four cisplatin-containing doublet combinations in stage IVB, recurrent, or persistent cervical carcinoma: a gynecologic oncology group study. J Clin Oncol.

[11] Harari PM. Epidermal growth factor receptor inhibition strategies in oncology. 2023. www.endocrinology-journals.org. 10.1677/erc.1.00600 15613446

[12] Normanno N, De Luca A, Bianco C, Strizzi L, Mancino M, Maiello MR (2006). Epidermal growth factor receptor (EGFR) signaling in cancer. Gene.

[13] Sigismund S, Avanzato D, Lanzetti L (2018). Emerging functions of the EGFR in cancer. Mol Oncol.

[14] Paez JG, Jänne PA, Lee JC, Tracy S, Greulich H, Gabriel S (2004). EGFR mutations in lung cancer: correlation with clinical response to gefitinib therapy. Science.

[15] Jiang H (2009). Overview of gefitinib in non-small cell lung cancer: an Asian perspective. Jpn J Clin Oncol.

[16] Kris MG, Natale RB, Herbst RS, Lynch TJ, Prager D, Belani CP (2003). Efficacy of gefitinib, an inhibitor of the epidermal growth factor receptor tyrosine kinase, in symptomatic patients with non-small cell lung cancer: a randomized trial. JAMA.

[17] Tang X, He J, Li B, Zheng Y, Li K, Zou S (2019). Efficacy and safety of gefitinib in patients with advanced head and neck squamous cell carcinoma: a meta-analysis of randomized controlled trials. J Oncol.

[18] Goncalves A, Fabbro M, Lhommé C, Gladieff L, Extra JM, Floquet A (2008). A phase II trial to evaluate gefitinib as second- or third-line treatment in patients with recurring locoregionally advanced or metastatic cervical cancer. Gynecol Oncol.

[19] Rawluk J, Waller CF (2018). Gefitinib. Recent Results Cancer Res.

[20] Zheng J, Yu J, Yang M, Tang L (2019). Gefitinib suppresses cervical cancer progression by inhibiting cell cycle progression and epithelial–mesenchymal transition. Exp Ther Med.

[21] Soonthornthum T, Arias-pulido H, Joste N, Lomo L, Muller C, Rutledge T (2011). Epidermal growth factor receptor as a biomarker for cervical cancer. Ann Oncol.

[22] Noordhuis MG, Eijsink JJH, Ten Hoor KA, Roossink F, Hollema H, Arts HJG (2009). Expression of epidermal growth factor receptor (EGFR) and activated EGFR predict poor response to (Chemo)radiation and survival in cervical cancer. Clin Cancer Res.

[23] Tsuda N, Watari H, Ushijima K (2016). Chemotherapy and molecular targeting therapy for recurrent cervical cancer. Chin J Cancer Res.

[24] Sharma DN, Rath GK, Julka PK, Gandhi AK, Jagadesan P, Kumar S (2013). Role of gefitinib in patients with recurrent or metastatic cervical carcinoma ineligible or refractory to systemic chemotherapy: first study from Asia. Int J Gynecol Cancer.

[25] Benson RB, Pathy S, Kumar L, Mathur S, Dadhwal V, Mohanti BK (2019). Locally advanced cervical cancer - neoadjuvant chemotherapy followed by concurrent chemoradiation and targeted therapy as maintenance: a phase II study. J Cancer Res Ther.

[26] Schwartz LH, Litière S, De Vries E, Ford R, Gwyther S, Mandrekar S (2016). RECIST 1.1-Update and clarification: from the RECIST committee. Eur J Cancer.

[27] Cancer Institute N. Common Terminology Criteria for Adverse Events (CTCAE) Common Terminology Criteria for Adverse Events (CTCAE) v5.0. 2017. https://www.meddra.org/.

[28] Singh MP, Chauhan AS, Rai B, Ghoshal S, Prinja S (2020). Cost of treatment for cervical cancer in India. Asian Pac J Cancer Prev.

